# Characterizing open and avoidant communication in parents’ caregiving experiences of adolescents and young adults (AYAs) living with blood cancer: Linking communication and psychosocial outcomes

**DOI:** 10.1017/S1478951525101417

**Published:** 2026-01-09

**Authors:** Emma G. Bryan, Kevin B. Wright, Samantha Reese, M. Devyn Mullis, Carma L. Bylund, Maria Sae-Hau, Elisa S. Weiss, Joanne P. Lagmay, Carla L. Fisher

**Affiliations:** 1 Department of Health Outcomes and Biomedical Informatics, University of Florida, Gainesville, FL, USA; 2 Department of Communication, George Mason University, Fairfax, VA, USA; 3 Cancer Control and Population Sciences Program, University of Florida Health Cancer Center, University of Florida, Gainesville, FL, USA; 4 Blood Cancer United, Washington, DC, USA; 5 Department of Pediatrics, Pediatric Hematology/Oncology, University of Florida, Gainesville, FL, USA

**Keywords:** Parent caregivers, adolescent and young adults, hematologic malignancy, communication, openness

## Abstract

**Objectives:**

Open communication between parents and adolescents and young adults (AYAs) with blood cancer is key to managing cancer together. However, parents avoid difficult conversations about cancer care and lack support in navigating them. To inform a communication skills intervention to help parents of AYAs navigate challenging conversations in caregiving, this mixed-method study sought to identify difficult topics and better understand psychosocial factors associated with avoidant communication.

**Methods:**

Phase 1 involved 20 interviews with parents of AYAs with blood cancer (aged 15–29) to capture difficult conversations and factors that inform why they are challenging. Phase 2 surveyed 80 parents about openness, avoidance, and psychosocial outcomes.

**Results:**

In Phase 1, parents identified 5 challenging conversation areas: (1) expressing negative feelings; (2) discussing disease/care-related information; (3) addressing sexual health; (4) navigating triadic clinical interactions; and (5) talking about mortality. Parents described 3 interrelated factors that informed why these conversations were difficult: (1) lifespan/human development; (2) emotional/psychological well-being; and (3) relational-caregiving dynamics. Quantitative results (Phase 2) confirmed the same challenging conversation areas and extended them with an additional topic parents avoid: caregiver burden. Overall avoidance of these topics was associated with lower clinical communication skills and competence, less openness between parents and AYAs, reduced willingness to communicate about cancer, and greater parental distress. Avoidance of discussing caregiver burden and sexual health with their AYA was associated with higher burden. Younger parents reported higher overall avoidance compared to older ones. Hispanic/Latino parents reported higher overall avoidance than non-Hispanic/Latino. Parents without a high school degree had higher scores for avoiding treatment discussions compared to parents with higher education levels.

**Significance of results:**

Findings highlight the need for supportive care interventions that strengthen parent caregivers’ communication skills. This study also provides a roadmap of key content to include, ensuring communication skills interventions are relevant and impactful.

## Introduction

Parents caring for adolescents and young adults (AYAs) with cancer play a primary role in care management and psychosocial support from diagnosis through survivorship (Berger et al. 2023; McLoone et al. 2023; Nightingale et al. 2022). As parents manage logistical, emotional, and financial pressures of caregiving, they encounter significant psychological distress and burden (Bilodeau et al. 2018; Hoven et al. 2017; Norberg and Boman 2013; Tillery et al. 2020). Parents’ outcomes may also impact AYAs as caregivers’ distress is interrelated with patients’ distress (Hodgson et al. 2021; Juth et al. 2015; Thompson et al. 2021), adjustment, and internalizing problems (Neves et al. 2023). Given these implications, research on and supportive care for parents is essential (Reuvers et al. 2023; Melguizo-Garin et al. 2022) but remains scarce (Berger et al. 2023; Pettitt et al. 2024).

The limited research on parents’ needs highlights relational tensions with AYAs in cancer caregiving (Pettitt et al. 2024; Zapata et al. 2023), contributing to developmental struggles (Barbot et al. 2022; Jones et al. 2011; Williams et al. 2014) and conflicts over care, control, and privacy (Chiara Magni et al. 2015; Phillips et al. 2017). Open communication is key to navigating these challenges (Ell 1996; Son and Kim 2024) and is linked to better psychological and relational outcomes across cancer caregiver groups, including spouses (Goldsmith et al. 2016; Manne et al. 2006; Traa et al. 2015), adult children, and parents of adult children (Campbell-Salome et al. 2022; Fisher et al. 2023, 2017; Wright et al. 2023). Yet families often avoid difficult care conversations and believe doing so protects themselves or their loved one (Fisher 2014; Fisher et al. 2017; Keitel et al. 1990). AYAs are especially avoidant – whether living with cancer themselves or a parent with cancer (Fisher et al. 2017) – and use avoidance to cope with distress or uncertainty (Fisher et al. 2017). Unlike openness, avoidance is not only linked with increased distress in caregivers and patients, (Fisher et al. 2017; Manne et al. 2006) but also poorer patient health outcomes, like fatigue and pain (Fisher et al. 2017). Research using family systems and lifespan frameworks demonstrates that families can be characterized by how openly they typically communicate – a pattern of communication that develops within their family environment and will ultimately inform how they cope with illnesses like cancer (Campbell-Salome et al. 2022; Koerner and Fitzpatrick 2002). Families with a more open communication pattern report better family functioning and interrelated health outcomes when navigating cancer (Campbell-Salome et al. 2022; Fisher et al. 2017).

Parents need help facilitating openness with their AYAs across the cancer trajectory to promote better health outcomes. As Rolland’s (2005) family system genetic illness model advocates, family-centered approaches that are targeted based on the relationship, developmental/lifespan factors, and disease are needed to meet caregivers’ distinct needs. Grounded in a family systems-lifespan approach, a recent web-based cancer caregiver intervention (*Healthy Communication Practice*) helps adult–child caregivers develop open communication skills to navigate challenging topics in online, clinical, and family care domains (Bylund et al. 2022; Fisher 2025; Fisher and Campbell-Salome 2025). The intervention was feasible and acceptable for adult–child caregivers of diagnosed parents with blood cancer (Bylund et al. 2022) and efficacious in reducing caregivers’ distress and improving their communication skills (Fisher 2025). To adapt this intervention for parents of AYAs with hematological malignancies, we investigated the following questions using a 2-phase, mixed-method design:

Phase 1

RQ1: What do parents identify as challenging conversations with their diagnosed AYA?

RQ2: What underlying factors do parents describe that inform why these conversations are challenging?

Phase 2

RQ3: What topics do parents report as most challenging and potentially avoided when talking with their AYAs?

RQ4: What communication factors – specifically, openness, willingness to communicate about health, and clinical communication competence – are associated with parents’ avoidance of cancer-related communication?

RQ5: What individual characteristics contribute to parents’ avoidant communication patterns?

RQ6: What psychosocial outcomes are associated with parents’ communication patterns?

### Methods

This study was part of a 2-phase, mixed-method exploratory design (Creswell 2014) to adapt a cancer caregiver communication skills intervention. This approach was in line with the design used to develop the original intervention: Phase 1 involved a qualitative approach (interview methodology) followed by Phase 2, which employed a quantitative method (survey) to build upon the qualitative findings (Bylund et al. 2022; Campbell-Salome et al. 2022; Fisher et al. 2021a, 2021b; Kastrinos et al. 2022).

### Recruitment and inclusion criteria

For interviews (Phase 1), caregivers were recruited via email through Blood Cancer United, formerly The Leukemia & Lymphoma Society, via their constituent database. Postings were also used with advocacy organizations. Inclusion criteria were (1) a parent caring for an adolescent aged 15–18 or emerging adulthood (EA) aged 19–29 diagnosed with a blood cancer >3 months prior and in active treatment or within 2 years since treatment ended, and (2) English-speaking. Interested parents contacted a coordinator via a link to screen for eligibility and interview scheduling. For survey recruitment (Phase 2), the same approach was used with 1 recruitment channel added (patient electronic health record system). Inclusion criteria were also expanded to parents of AYAs diagnosed or relapsed in <5 years.

### Phase 1: Interview method and procedures

For RQs1 and 2, a semi-structured, in-depth interview guide was developed by authors with expertise in caregiving [CLF, CLB], blood cancer [JPL, MSH, ESW], as well as family [CLF, MDM, CLB], clinical [CLF, CLB], and online [CLB] communication. Interviews were audio-recorded and conducted by research assistants. Questions explored caregiving in online, clinical, and family contexts to understand challenges and communication skill needs. Interviews lasted approximately 92 min (range, 25–200 min). Transcription resulted in 472 single-spaced pages of data.

### Phase 2: Survey measures

For RQs3–6, caregivers responded to an online survey upon receiving a link from a research assistant. The survey consisted of validated scales to measure communication patterns and psychosocial outcomes, specifically avoidant communication, open communication, willingness to communicate about health (cancer), clinical communication competence, caregiving burden, and psychological distress.

#### Avoidant communication

Avoidant communication was measured using the Cancer-Topic Avoidance (CTA) scale (Donovan-Kicken and Caughlin 2011; Yu and Sherman 2015) (α = .94), which was developed with spousal cancer caregivers and patients. It has been adapted and applied to multiple types of cancer caregivers and relationships (Fisher et al. 2023; Wright et al. 2023). It addressed 5 care-related topics: mortality, caregiver burden, negative feelings, sexual health, and treatment. Sexual health was slightly modified based on Phase 1 results to target parents. Items were rated on a 5-point scale from (1) “Strongly disagree” to (5) “Strongly agree,” with higher scores indicating more avoidance.

#### Open communication

Open communication was measured using a subscale (conversation orientation: 6-items) from the short form of the Revised Family Communication Pattern scale, which measures how open parents and AYAs typically communicate in their relationship (Fisher et al. 2023; Wilson et al. 2014) (α = .77). Questions in the subscale include “I encourage my child to express his/her feelings.” Items were rated on a 5-point scale from (1) “Strongly disagree” to (5) “Strongly agree.” Subscale reliability was good.

#### Willingness to communicate about health – cancer

Parents’ willingness to talk about cancer with their AYA was captured with the 6-item Willingness to Communicate about Health scale (Wright et al. 2007) (α = .76), which has been adapted for various health contexts. The scale was slightly modified to target parents’ communication with their child and others about caregiving. Items were rated on a 5-point scale from (1) “Strongly disagree” to (5) “Strongly agree” with items including, “I’m comfortable talking about caregiving issues with my child with a blood cancer.”

#### Clinical communication competence

The 11-item Patient Report of Communication Behavior (PRCB-CG; Bylund et al. 2010) was used to measure parents’ perceptions of their communication skills when talking to their child’s clinicians (α = .85). Items were rated on a 5-point scale from (1) “Never” to (5) “Always” with items including “I tell my doctors when I want more information about something.”

#### Caregiver burden

The 12-item Zarit Burden Interview-Short Form (Bedard et al. 2001) captured parents’ perceived caregiving burden (α = .85). Items were rated on a 5-point scale from (1) “Never” to (5) “Nearly Always” with items including, “How often do you feel strained when you are around your child?” Higher scores indicated increased burden.

#### Psychological distress

The 37-item Profile of Moods State (POMS) short form-cancer scale was used to measure parents’ distress (Baker et al. 2002; Kim et al. 2007) (α = .92). Parents rated their feelings during the past week using a 5-point scale from (1) “Not at all” to (5) “Extremely.” Items were grouped into mood state scores for depression, anger, tension, confusion, fatigue, and vigor.

### Phase 1: Interview data analysis (RQs1 and 2)

Transcripts were thematically analyzed using a constant comparative method approach (Strauss and Corbin 1998) by inductively coding for patterns, collapsing codes into categories to identify themes, and axial coding for thematic properties. A multi-coder approach was used. One coder [SR] began inductive analysis of a subset of transcripts, meeting with a qualitative and family cancer caregiving expert [CLF] who guided and refined analysis for codebook development. Transcripts were added using the same process. A qualitative expert [MDM] used a preliminary codebook to deductively and inductively analyze the dataset and finalize a codebook with the senior author/expert [CLF]. Consensus meetings were held to refine typologies. Criteria for thematic saturation (Owen 1984) and verification techniques promoted rigor (multiple coders, content and methods experts, expert overseeing analysis, triangulating EA with adolescent caregivers) (Morse et al. 2002). Regarding the extent of thematic saturation, all themes were reported by 35–75% of parents.

### Phase 2: Survey data analysis (RQs3–6)

Pearson’s correlations were computed using SPSS 24 to assess relationships between topic avoidance and communication factors and health outcomes. Independent *t*-tests and 1-way ANOVAs were done to determine the relationships between avoidance and individual characteristics.

## Results

Participant demographics for the interview phase are reported in Table 1.

**Table 1.** Parent caregiver interview participants’ and their AYAs’ demographics (*N* = 20)

Characteristic	*n* (%)	Mean (SD)	Min–Max
Age			
Caregiver		52 (6.76)	38−69
Patient age at diagnosis		19.9 (3.99)	15−28
Patient Developmental Group			
Adolescent	10 (50)		
Emerging adulthood	10 (50)		
Caregiver Gender			
Female	19 (95)		
Male	1 (5)		
Patient Gender			
Female	5 (25)		
Male	15 (75)		
Caregiver Race/Ethnicity			
White, non-Hispanic	16 (80)		
White, Hispanic/Latino	2 (10)		
African American, non-Hispanic	1 (5)		
African American, Hispanic/Latino	1 (5)		
Caregiver Employment Status			
Employed, full-time	10 (50)		
Employed part-time	5 (25)		
Not employed/Retired	5 (25)		
Patient Diagnosis^a^			
Leukemia	15 (75)		
Lymphoma	5 (25)		
Patient Diagnosis Subtype			
Acute myeloid leukemia	4 (20)		
Acute lymphoblastic leukemia	9 (45)		
T-cell leukemia	1 (5)		
Hodgkin’s lymphoma	4 (20)		
Non-Hodgkin’s lymphoma	1 (5)		
Diffuse large B-cell lymphoma	1 (5)		

a One AYA was diagnosed with both leukemia and lymphoma.

### Interview findings: Challenging conversations (RQ1)

Parents described 5 challenging conversation areas: (1) expressing negative feelings; (2) discussing disease/care-related information; (3) addressing sexual health; (4) navigating triadic clinical interactions; and (5) talking about mortality. Parents’ narratives illustrate the complexity they face communicating about these issues, which they described contributing to tension, conflict, and avoidance. Each topic area (i.e., theme) is defined with properties (italicized) to characterize the challenging conversation areas.

#### Negative feelings

Parents emphasized challenges related to sharing negative feelings or distress (e.g., sadness, anxiety, fear). This included *parents’ or AYAs’ negative affect*: “I’m just scared out of my mind that it’s going to come back.… When I look at [him] sometimes I just cry.… He’s like, ‘Why are you crying, Mom?’” (Mother, EA). Similarly, parents struggled with AYAs’ negative affect: “It’s like there was this underlying anger, and it came out towards us, but … really, she was angry at life” (Mother, EA). Parents also identified challenges addressing *AYAs’ emotional/mental reactions to treatment*. Treatment or medication side effects contributed to AYAs’ negative affect and, at times, uncharacteristic behavior (e.g., low mood, aggression, personality changes): “Certain medications just made him grumpy, and he was quick to acknowledge that. He’s like, ‘I’m so impatient right now’”(Mother, Adolescent).

#### Disease-/Care-related information

Parents described difficulty talking about disease- or care-related information with their AYAs, much of which centered around treatment. Parents sometimes avoided discussions or withheld information perceived as *negative or scary*, such as prognosis, treatment decisions, or other AYAs’ disease/treatment experiences: “I wouldn’t let any negativity come near us, me or [son].… I’ve shielded him from all of that” (Mother, Adolescent). Parents also found it difficult *to gain or give additional care information*, like when parents gave AYAs treatment- or care-related advice or suggestions:

We talked with three nutritionists. That is an area that I really think was a huge source of conflict for us.… It wasn’t just calories he needed, but he needed nutrients … There were times that we verbally disagreed, and he was angry with me for pushing this on him. (Mother, EA)

#### Sexual health

Parents struggled talking about AYAs’ sexual health. *Onco-fertility* discussions could be particularly uncomfortable for parents and AYAs:

He’s 17. He’s been raised in a church. He’s been raised in a biblical family his entire life. Do we freeze your sperm? That was horrible. Do you want us to freeze your sperm? And that was like day 12 or 13 of his diagnosis. (Mother, Adolescent)

Parents also found it challenging to discuss *AYA’s safe sex practices*, which was necessary during treatment: “I was a little careful when I brought it up because maybe he doesn’t want to share his sex life with me. So, I had to be respectful” (Mother, EA).

#### Triadic clinical interactions

Parents encountered challenges interacting with AYAs and their healthcare team. During medical encounters, parents were met with challenges when they *shared information the AYA withheld or didn’t disclose* or, as a parent explained, when they “told [the] truth” or were “ratting [AYA] out.” Parents communicated information to ensure providers had knowledge they felt was necessary for care:

Me not asking permission to share things or … telling the truth … [like doctor asks] “Have you been drinking a lot?” He’s like, “Yeah”, and I’m like, “Nope!” … That’s just me being honest because it’s in his body and so being too honest. And then he’ll just glare at me, which I don’t care about. That is tension, but the oncologist or the PA now know. But it’s funny because I can feel them looking back and forth at us like, “Okay …” It’s awkward for them.… They just need to know. I don’t know. So, being too honest has created tension. (Mother, EA)

Additionally, parents described challenging conversations when there was *discordance between parent and AYA about what to discuss.* This occurred when parents wanted to address something with clinicians that related to decision making and being prepared, which conflicted with AYAs’ information preferences. In part, this intersected with disease-related information:

She had radiation [soon]. I emailed the social worker, and I was kind of upset because nobody mentioned her losing [her hair]. As a young adult, the hair is a big thing. It’s very difficult to be bald as a woman in college and at work.… [Daughter] wouldn’t ask the question and got mad at me for asking her to ask the question.… She [said], “I swear, it’s almost like you want me to lose my hair.” … Ten days after her radiation her hair just sloughed off. (Mother, EA)

#### Mortality

Conversations about death were particularly challenging. This included fears related to *survival/prognosis* (e.g., survival/death rates) – a concern for AYAs and parents. Parents admitted avoiding the topic or buffering conversations with positivity:

We just tried to be positive with him.… [We would say] “Let’s not think about that. Let’s just be positive, and we’re going to hope for the best.” … We had a lot of difficult conversations with him because he was really worried that he could die. (Mother, Adolescent)

One parent also experienced struggles discussing *end-of-life decisions*:

He talked about things like what he would want if he were to pass away.…He got to see [his aunt before she died.] … That made him think … if I’m in that position, I don’t want a bunch of people just sitting there with me waiting for me to die, and so he did think through some things and we talked through some things like that.… It’s hard to have those conversations, because you just don’t want it to happen. (Mother, EA)

### Interview findings: Underlying factors (RQ2)

Parents described 3 underlying, interrelated factors that informed why these conversations were challenging and hard to navigate: (1) lifespan/human development; (2) emotional/psychological well-being; and (3) relational-caregiving dynamics.

#### Lifespan/human development

Given AYAs were facing a life-threatening disease early in the lifespan, parents shared that addressing certain topics (e.g., mortality, triadic clinical interactions; sexual health) felt atypical or non-normative, which made these conversations especially challenging. AYAs were still developing maturity and independence, and certain issues were outside the bounds of the typical trajectory of adolescence and young adulthood:

You would never think that you would have to discuss fertility issues with your 15-year-old. That’s a really backwards thing. That’s a really upside down situation. That shouldn’t really happen.… You should have puberty talk with them and stuff like that for sure. And sex talk with them and all of that, which, you know, we basically had already done. But having to talk about something that most teenagers don’t have to experience, that’s just really different. (Mother, Adolescent)

Lifespan factors sometimes intersected with relational dynamics. For instance, topics like sexual health/onco-fertility, that were necessary to broach for decision making felt like an invasion of privacy as their parent or inappropriate this early in life: “[I said] ‘Your physical life is your business, and I don’t need to know anything about it. If you didn’t have cancer, I would know nothing and probably wouldn’t want to know anything’” (Mother, EA).

#### Emotional/psychological well-being

Parents shared it was important to consider AYAs’ emotional well-being when deciding whether to approach certain conversations. For instance, AYAs’ emotional state sometimes informed how they reacted to the conversation (e.g., disease-related information, triadic clinical conversations): “It disrupts their personality so badly.… They’re saying things to you that she would have never said as a pre-cancer person, and it’s that toxicity and all the medications. That’s very difficult” (Mother, EA). Parents also wanted to prioritize their AYAs’ psychological state and shield them from more distress. Their desire to protect AYAs’ psychological well-being took precedence over talking about or pursuing some topics (e.g., disease/care-related information, negative feelings, mortality): “I didn’t hit him on every little thing because I didn’t think it helped. It would have just added worry.… It just wasn’t necessary” (Mother, EA).

#### Relational-caregiving dynamics

Parents illustrated complex relational dynamics in their role as both parent and cancer caregiver, which could challenge typical parent–child dynamics or boundaries related to control, independence, and privacy when certain challenging conversations emerged (e.g., triadic medical interactions, disease-related information). At times the need for care conversations conflicted with parents’ perceived gendered parenting role:

As a dad, whether she was going through this illness or not, I’m the dad. I’m always uncomfortable hearing about my daughter’s sexual life. Like, No, you don’t do that stuff! You’re only 25 and living with this guy for five years, who, by the way, we love [but] you don’t do that stuff. (Father, EA)

These dynamics were embedded within the typical challenges of parenting an AYA – a time marked by a renegotiation of boundaries and increasing independence:

For her to feel like we’re spoon-feeding her stuff, it was very difficult for her.… What’s the best way to make them feel empowered even though they have some issues right now that are temporary? That was very difficult. I felt like I was constantly treading a line down to like, should I get her water bottle? … She would just snap at me, “I know I can get water when I need water.” … It was very difficult because her independence was just clipped, just like that, one day, and she was ready—ready to go. (Mother, EA)

### Phase 2 – Survey findings

Survey participant demographics are in Table 2.

**Table 2.** Parent caregiver survey participants’ and their AYAs’ demographics (*N* = 80)

Characteristic	*N*	%
Parent Caregiver Age^a^		
30–49 years	26	34.2
50–64 years	50	65.8
Patient Developmental Group		
Adolescent	25	31.25
Emerging adulthood	55	68.8
Caregiver Gender		
Female	69	86.3
Male	8	10
Nonbinary	1	1.3
Other	1	1.3
Prefer not to say	1	1.3
Patient Gender		
Female	26	32.5
Male	51	63.8
Nonbinary	2	2.5
Other	1	1.3
Caregiver Ethnicity		
Non-Hispanic/Latino	70	87.5
Hispanic/Latino	10	12.5
Caregiver Race^b^		
White	61	79.2
Black or African American	10	13.0
Asian	6	7.8
Caregiver Employment Status		
Employed, full-time	43	53.8
Employed part-time	15	18.8
Not employed/Retired	22	27.6
Caregiver Education Level		
Less than high school diploma	3	3.8
High school graduate or equivalent	9	11.3
Some college, no degree	10	12.5
Associate degree	13	16.3
Bachelor’s degree	19	23.8
Professional or graduate degree	20	25.0
Doctorate	6	7.5
Patient Diagnosis ^1^		
Leukemia	49	61.3
Lymphoma	31	38.8
Disease Subtype		
Acute lymphoblastic leukemia	27	33.8
Acute myeloid leukemia	9	11.3
Acute promyelocytic leukemia	4	5.0
Chronic lymphocytic leukemia/Small cell Lymphocytic lymphoma	1	1.3
Chronic myeloid leukemia	5	6.3
Other type of leukemia	3	3.8
Hodgkin’s lymphoma	19	23.8
Diffuse large B-cell lymphoma	8	10.0
Other type of lymphoma	4	5.0

a Data are missing from 4 participants.

b Data are missing from 3 participants.

#### Survey findings: Avoided topics (RQ3)

Survey results validate and extend interview findings. Parents reported the same topics as challenging to discuss with their AYAs (e.g., mortality, sexual health, feelings, disease information–specifically treatment). They also identified an additional topic: caregiving burden (see Table 3). Parents rated mortality as most challenging, followed by sexual health, feelings, burden, and treatment.

**Table 3.** Parent caregiver topic avoidance scores (*N* = 80)

Topic	Mean	Standard deviation
Mortality	3.56	.87
Sexual health	3.28	1.17
Feelings	2.93	1.12
Burden	2.73	.92
Treatment	2.65	1.17

#### Survey findings: Avoidance and communication factors (RQ4)

Parents’ avoidance of cancer-related topics correlated with communication factors. Greater avoidance was significantly associated with less communication competence, including less openness, reduced comfort talking about cancer (i.e., willingness), and lower clinical communication skills (see Table 4).

**Table 4.** Correlation between parents’ topic avoidance and communication factors (*N* = 80)

Topic	Willingness to communicate about health (cancer)	Clinical communication competence	Openness
Mortality	−.24*	−.29*	−.37*
Sexual Health	−.21	−.26*	−.19
Feelings	−.18	−.30**	−.36**
Burden	−.28*	−.22*	−.25*
Treatment	−.25*	−.23*	−.19
Overall Avoidance	−.28*	−.32*	−.32*

* Correlation is significant at *p* < .05.

** Correlation is significant at *p* < .001.

#### Survey findings: Avoidance and demographic factors (RQ5)

Several characteristics informed parents’ communication patterns. Younger parents (age 30–49) reported more avoidance (*M* = 3.32; *SD* = .74) than older parents (aged 50–64) (*M* = 2.82; *SD* = .86), *t* = 2.484, *p* = 0.01, and Hispanic/Latino parents showed higher avoidance (*M* = 3.66; *SD* = .63) compared to non-Hispanic/Latino parents (*M* = 2.96; *SD* = .82), *t* = 2.62, *p* = .01. Table 5 compares avoidance scores for each topic based on parent age and ethnicity. Although overall avoidance did not vary by education level, avoidance of cancer treatment discussions differed significantly by education, *F*(6, 79) = 2.757, *p* = 018. Post hoc comparisons using the LSD test revealed that parents who did not have a high school degree had higher scores for avoiding treatment discussions compared to parents with higher education levels. No significant differences were found in avoidance scores (overall or by topic) based on race or gender.

**Table 5.** Differences in avoidance (CTA) based on parent demographic factors (*N* = 80)

Demographic factors	Topic	Group 1 (*M*, SD)	Group 2 (*M*, SD)		
Parent age-group		Younger (*n* = 26) (age 30−49)	Older (*n* = 50) (age 50−69)	Test statistic	*P*
	Treatment	3.07 (1.07)	2.36 (1.14)	*t* = 2.6*	.01
	Sexual health	3.63 (.86)	3.06 (1.27)	*t* = 2.06*	.04
	Mortality	3.67 (.80)	3.47 (.91)	*t* = .90	.37
	Burden	3.04 (.82)	2.51 (.89)	*t* = 2.496*	.01
	Feelings	3.20 (.97)	2.73 (1.16)	*t* = 1.762	.08
**Ethnicity**		**Hispanic (*n* = 10)**	**Non-Hispanic (*n* = 70)**		
	Treatment	3.62 (1.06)	2.51 (1.12)	*t* = 2.949**	.002
	Sexual health	3.60 (.77)	3.24 (1.22)	*t* = .92	.36
	Mortality	4.16 (.50)	3.47 (.87)	*t* = 2.40*	.02
	Burden	3.34 (.90)	2.64 (.91)	*t* = 2.280*	.013
	Feelings	3.44 (.93)	2.86 (1.13)	*t* = .92	.13

#### Survey findings: Avoidance, openness, and psychosocial outcomes (RQ6)

Intersections were found between communication (openness and avoidance) and psychosocial outcomes (caregiver distress and burden). Greater avoidance was associated with increased overall distress, as well as lower vigor and higher levels of tension, anger, and confusion. While overall avoidance was not significantly correlated with burden, avoidance of talking about certain topics – specifically caregiver burden and sexual health – showed significant associations with higher burden. See Tables 6 and 7 for additional correlations between these psychosocial outcomes and each topic. Regarding openness, no significant correlations with distress or burden were found, though a trend toward significance was observed for burden (*r* = .−19, *p* = .088).

**Table 6.** Correlations between avoidance (CTA) and distress (POMS) among parents (*N* = 80)

Topic	Vigor	Depression	Anger	Tension	Confusion	Fatigue	POMS total
**Mortality**	−.28*	.28*	.17	.30**	.42**	.18	.33**
**Sexual health**	−.26*	.05	.19	.16	.19	.16	.21
**Feelings**	−.11	.13	.21	.15	.16	.01	.14
**Burden**	−.23*	.16	.32**	.14	.16	.05	.20
**Treatment**	−.14	.15	.06	.16	.16	.01	.12
**Overall avoidance**	−.24*	.18	.23*	.21	.26*	.09	.24*

* Correlation is significant at *p* < .05.

** Correlation is significant at *p* < .01.

**Table 7.** Correlations between CTA and Burden (ZBI) among parents (*N* = 80)

Topic	Caregiver burden
**Mortality**	.13
**Sexual health**	.25*
**Feelings**	.16
**Burden**	.23*
**Treatment**	−.03

* Correlation is significant at *p* < .05.

** Correlation is significant at *p* < . 001.

## Discussion

These findings provide evidence for prioritizing supportive care interventions addressing parent caregivers’ communication skill development to enhance psychosocial outcomes. While clinicians’ communication skills have been prioritized in medical education (Smith et al. 2020), emphasizing family caregivers’ communication skills is largely absent in oncology care. Our findings highlight the need for communication support for parent caregivers of AYAs with blood cancer to promote better psychosocial outcomes and care.

Our findings showed that when parents lack communication skills, they experience poorer psychosocial outcomes. Parents with less competence in communicating openly with their AYA about specific topics – such as caregiver burden and sexual health – report more burden, and those who avoid talking about care in general report higher overall psychological distress. These findings emphasize the importance of prioritizing parents’ communication skill development in AYA oncology care and improving caregivers’ quality of life, as caregiver outcomes are interrelated with their diagnosed AYAs (Thompson et al. 2021).

While findings from both study phases demonstrate that caregiving parents of AYAs would collectively benefit from open communication skill development, the survey findings highlight 3 demographic factors that give further insight into parents with a heightened need. Both younger parents (>49 years) and parents who identify as Hispanic reported more avoidance overall, and parents with lower education (<high school diploma) were especially avoidant of cancer treatment discussions. Age may, in part, be tied to less competence in talking openly for parents, whereas a lower education level could be related to lower health literacy, which can contribute to avoidance or silence in cancer conversations (Holden et al. 2021). With regard to ethnicity, a recent systematic review showed that Hispanic families facing cancer tend to be less open and more avoidant of cancer disclosures in comparison with other racial-ethnic minority groups, as they do not want to cause family members burden, worry, or distress (Huang et al. 2022). Yet, at the same time, their avoidance has been linked to higher levels of individual and relational stress as well as poorer family functioning. Collectively, these findings suggest that caregiving parents of AYAs who identify as Hispanic, do not have at least a high school level of education, and who are under the age of 49 may have a heightened need for learning how to communicate more openly.

Further, the results provide a roadmap of content to prioritize in supportive care interventions when helping parents develop communication skills. First, parents need communication skills in 2 areas: facilitating openness with AYAs and communicating with clinicians. Second, the mixed-methods findings provide strong evidence of the most challenging topics that parents avoid and may create tension with their AYAs. Three of these topics seem particularly important to emphasize given links to parents’ burden and distress, specifically mortality (e.g., survival rates, prognosis, relapse fears, end-of-life); sexual health (e.g., onco-fertility, sexual activity); and negative feelings (e.g., fears, sadness, anger). Third, parents’ narratives could be used to illustrate the challenging nature of these topics while teaching communication skills. These narratives also help characterize the underlying factors that make these conversations distinctively complex in AYA caregiving (AYAs’ age, emotional well-being, relational dynamics).

Finally, the quantitative results revealed parents need help talking about their care burden. Healthcare professionals, particularly nurse navigators and oncology social workers, interact with parents frequently and can be key sources of psychosocial support to AYAs and parents (Bouchard et al. 2023; Gise and Cohen 2022). Integrating clinical time to assess parents’ psychosocial needs would create opportunities for parents to openly share their burden-related concerns and alleviate distress with a health professional. Our findings support the argument that caregivers be integrated into oncology care as patients themselves so that they can be assessed for and connected to psychosocial support. This is further heightened by the reality that family cancer caregivers of AYAs report significant burden and psychological distress including more anxiety than patients (Reuvers et al. 2023).

A few limitations should be considered for future studies. Our sample was largely white, non-Hispanic with more mothers. Oversampling men using strategic messaging could ensure fathers’ perspectives are more represented (Yaremych and Persky 2023). Future studies could also capture AYAs’ perspectives. Psychosocial support that attends to both parents’ and AYAs’ communication skill development may be more impactful in promoting better psychosocial outcomes for both parents and AYAs. Finally, future studies can explore whether other topics pose challenges for parents and AYAs living with solid tumor cancers.
